# Thyrotoxic Periodic Paralysis- A Diagnostically Challenging Rare Clinical Entity

**DOI:** 10.7759/cureus.11804

**Published:** 2020-11-30

**Authors:** Naga Vaishnavi Gadela, Anantha Sriharsha Madgula, Dimitrios Drekolias, Mahati Paravathaneni, Viraj Modi

**Affiliations:** 1 Internal Medicine, University of Connecticut, Farmington, USA; 2 Internal Medicine, Mercy Catholic Medical Center, Darby, USA

**Keywords:** thyroid, periodic paralysis, hypokalemia

## Abstract

Thyrotoxic periodic paralysis (TPP) is characterized by a classic triad of muscle paralysis, hypokalemia, and hyperthyroidism. The underlying thyroid disorder is often very subtle making it challenging to recognize TPP. It is a completely reversible condition if diagnosed early; however, it is associated with fatal outcomes if delayed.

## Introduction

Thyrotoxic periodic paralysis (TPP) is characterized by a triad of muscle paralysis, intracellular sequestration of potassium without a total body deficit, and hyperthyroidism [1]. Diagnosis of TPP is often delayed mainly because of its overlapping symptoms with other neurological disorders, the subtleness of the underlying thyrotoxic state, and also unawareness of this condition. TPP has a prevalence of 0.1 to 0.2% of people with hyperthyroidism in the North American population [1]. It is more common in Asian countries where 2% of patients with thyrotoxicosis reportedly develop TPP [2,3]. The presence of different genetic subtypes like HLA-DRw8 in Japanese people, HLA-A2, Bw22, Aw19, and B17 in Singapore Chinese, and B5 and Bw46 in Hong Kong Chinese, may make these patients susceptible to TPP [4]. Although thyrotoxicosis is more prevalent in women, TPP occurs predominantly in men, with a male to female ratio of 30:1 [5]. 

## Case presentation

A 46-year-old man with Graves’ disease, on methimazole and propranolol, presented to our hospital with an abrupt onset of weakness in his bilateral upper and lower extremities. The patient moved to the United States a few months ago from Puerto Rico, and he reported poor compliance with his medications. He denied any significant family history of thyroid/neurological disorders or electrolyte imbalances. His symptoms started abruptly with weakness in his upper and lower extremities at the same time, progressively worsening in a few hours to a point where he was unable to move. He denied bowel or bladder incontinence, recent infections, tick bites, back pain, fever, drug use, or trauma. Physical examination revealed symmetrical flaccid paralysis in bilateral upper and lower extremities, loss of deep tendon reflexes, without sensory or cranial nerve involvement, and no saddle anesthesia. Thyroid examination was unremarkable without significant enlargement/bruits. 

Laboratory investigations are outlined in Table 1.

**Table 1:** Laboratory investigations and reference ranges TSH: thyroid stimulating hormone, T3: triiodothyronine T4: thyroxine

INVESTIGATION		VALUE		REFERENCE RANGE	
Potassium		1.4mmol/liter		3.6-5.1mmol/liter	
Chloride		108mmol/L,		100-111mmol/L	
Magnesium		1.7mg/dl		1.8-3mg/dl	
Calcium		8.6mg/dl		8.9-10.4mg/dl	
Thyroid Stimulating Hormone		< 0.01uU/ml		0.35-4.94uU/ml	
TSH receptor antibody		29.10 IU/L		<=1.75 IU/L	
Total T3		209 ng/dl		48-178ng/dl	
Free T4		1.92ng/dl		0.8-2.8ng/dl	
Thyroid-stimulating immunoglobin		>500%		<=122%	

A diagnosis of Graves' disorder-induced thyrotoxic periodic paralysis (TPP) was made based on his clinical presentation, electrolyte abnormalities, and undetectable TSH levels. 

After the diagnosis of TPP was made, the patient was started on methimazole 20mg once, and methimazole 5mg thereafter, propranolol 20mg three times a day, potassium drip at 10mEq/hour with close monitoring of potassium every two hours, due to the risk of rebound hyperkalemia. The patient was monitored on telemetry. The patient slowly regained his muscle strength after the potassium came back to 2.2mmol/liter following 40mEq of potassium supplementation. The patient was discharged on methimazole and propranolol the following day, with close outpatient follow-up.

## Discussion

TPP occurs due to a thyrotoxic state, regardless of the etiology, severity, or duration of thyrotoxicosis [2]. However, Graves' disease is one of the most common underlying disorders in the majority of cases [6,7]. The pathogenesis is mostly attributed to the disruption of cellular transport mechanisms. The Na+-K+ ATPase channel maintains the transcellular distribution of electrolytes through the cell membrane and is affected by adrenergic stimulation. Thyroid hormone increases the tissue responsiveness to adrenergic stimulation, while increasing the Na+-K+ ATPase activity on the skeletal muscle cell through a genomic mechanism. This leads to translocation of potassium into the cell with resultant hyperpolarization, rendering the cells unexcitable, and hence develop muscle paralysis. This is supported by the finding that thyrotoxic patients with TPP have a higher pump activity compared to those without TPP [8]. Additional factors such as decreased efflux of potassium from the cell due to a loss of function mutation in the inward rectifying potassium channel in patients with TPP may also explain the hypokalemia [9]. 

The diagnostic approach begins with detailed history and physical examination. The muscle paralysis involves the proximal muscles of the lower limbs, and is characterized by acute areflexia or hyporeflexia. Mild myalgia and muscle stiffness can also be seen in some. However, sensory, bowel, and bladder function are not affected. It is important to differentiate TPP from a true neurologic condition such as cauda equina syndrome, transverse myelitis, or Guillain Barre syndrome. Rare cases of bulbar and respiratory weakness requiring ventilatory support, and fatal arrhythmias such as ventricular fibrillation, sinus arrest have also been reported secondary to TPP [6,10-12]. Although no obvious precipitant is identified in most cases, these attacks can be triggered by events that increase the release of epinephrine or insulin, both of which can cause movement of potassium into the cell, such as strenuous exercise or a high carbohydrate meal. 

Laboratory findings may include hypomagnesemia and mild hypophosphatemia along with hypokalemia. During an attack of paralysis, potassium levels can be as low as 1.1mmol/liter. The severity of the weakness often correlates with the degree of hypokalemia [13]. Most patients may have only mildly elevated thyroid hormone levels, and only a few of them have thyrotoxic symptoms. It is important to differentiate TPP from familial periodic paralysis (FPP) which presents similarly. FPP is an autosomal dominant condition with variable penetrance characterized by episodes of muscle paralysis. It is prevalent in young adults usually before puberty with a family history of similar paralytic episodes. It was also found that a urine calcium to phosphate ratio of more than 1:7 is a sensitive and specific marker to differentiate TPP from FPP [14].

Management of TPP includes correction of hypokalemia, and treatment of thyrotoxic state. Patients are usually given potassium repletion to hasten muscle recovery, however it is essential to recognize that hypokalemia is due to redistribution of potassium rather than true potassium loss. Hence there is a danger of rebound hyperkalemia on recovery. It is a common complication, especially if they have received >90mEq of potassium in the first 24 hours [6], whereas patients who have received <50mEq of potassium in the first 24 hours rarely develop rebound hyperkalemia. Therefore, slow repletion with lower doses may be effective in lowering the risk [15]. Nonselective beta-blockers such as propranolol prevent the intracellular shift of potassium by blunting the adrenergic stimulation of the Na+-K+ ATPase channel. Paralytic attacks do not recur once the patient achieves euthyroid state, hence definitive therapy is by controlling the underlying thyroid disorder with antithyroid medications, radioiodine therapy, or surgical thyroidectomy. It is also important to continue propranolol until a euthyroid state is obtained.

## Conclusions

Although TPP is traditionally more common in the Asian population, physicians should be vigilant because of rising immigrant populations. Understanding the pathogenesis can also prevent complications due to rebound hyperkalemia secondary to excessive potassium replacement. Early diagnosis is essential to prevent serious cardiopulmonary complications and the fatality associated with the syndrome. 
